# Rubber Crumb Infill in Synthetic Turf and Health Outcomes: A Review of the Literature on Polycyclic Aromatic Hydrocarbons and Metalloids

**DOI:** 10.3390/epidemiologia6010004

**Published:** 2025-01-25

**Authors:** Shamshad Karatela, Igor Popovic, Zahra Sobhani, Shiv Basant Kumar, Thava Palanisami, Li-Zi Lin, Gunther Paul

**Affiliations:** 1 Faculty of Medicine, Nursing and Health Sciences, School of Public Health and Preventive Medicine, Monash University, Melbourne, VIC 3004, Australia; 2 Faculty of Medicine, The University of Queensland, Brisbane, QLD 4006, Australia; i.popovic@uqconnect.edu.au; 3 Mater Research Institute, University of Queensland, Brisbane, QLD 4101, Australia; 4 Australian Institute of Tropical Health and Medicine, James Cook University, Townsville, QLD 4811, Australia; gunther.paul@jcu.edu.au; 5 UQ Spatial Epidemiology Laboratory, School of Veterinary Science, The University of Queensland, Gatton, QLD 4343, Australia; 6 Environmental Plastic Innovation Cluster (EPIC), Global Innovative Centre for Advanced Nanomaterials (GICAN), College of Engineering, Science and Environment, The University of Newcastle, Callaghan, NSW 2308, Australia; zahra.sobhani@newcastle.edu.au (Z.S.); shivbasantkumar@uon.edu.au (S.B.K.); thava.palanisami@newcastle.edu.au (T.P.); 7 Guangdong Provincial Engineering Technology Research Center of Environmental Pollution and Health Risk Assessment, Department of Occupational and Environmental Health, School of Public Health, Sun Yat-sen University, Guangzhou 510080, China; linlz@mail.sysu.edu.cn; 8 Aussie Ergonomics Pty Ltd., Mackay Harbour, QLD 4740, Australia

**Keywords:** artificial grass, synthetic turf, playground, crumb rubber, sportsground, polycyclic aromatic hydrocarbons, metal(loids)

## Abstract

Synthetic turf has become a popular alternative to natural grass due to low upkeep costs; however, its health impacts have not been clearly elucidated. This review examines and consolidates the existing literature on rubber crumb in infill in synthetic turf and its associated adverse health outcomes, along with recommendations for future research. A database search was conducted in PubMed, Web of Science, Scopus, Embase, and Google Scholar of studies on exposures to rubber crumb in infills in synthetic turf. The search focused on epidemiological and toxicological laboratory studies (including exposure simulation and animal studies), as well as government reports. Non-English studies and those addressing injuries (musculoskeletal and burn injuries) were not considered. Eighteen laboratory studies examined concentrations of PAHs found in synthetic turf rubber infill. The total level of PAHs detected in samples varied between 0.4 mg/kg and 3196 mg/kg. The PAH levels were influenced by the age of the synthetic turf, with the older synthetic surface fields containing lower concentrations (compared to newly laid turfs). Synthetic turfs composed of industrial rubber crumb infill also had a lower PAH composition relative to end-of-life tyre-derived infill. In the six studies that investigated the metal content and composition of rubber crumb infill, Aluminium (5382 mg/kg), Zinc (5165 mg/kg), and Iron (489.6 mg/kg) had the highest median concentrations. There were minor differences in heavy metal concentrations found in newly installed synthetic turf compared to older turfs and synthetic sporting fields exposed to direct sunlight (versus indoor fields). There were two epidemiological studies on synthetic turf rubber crumb infill (one ecological and one cross-sectional study), which found no significant associations between synthetic turf exposure and the incidence of leukemia, non-Hodgkin lymphoma, and Hodgkin lymphoma. Similarly, one metabolomic study of urine samples from athletes taken pre- and post-match on synthetic turf, and two studies simulating dermal, ingestion, and inhalation exposure concluded that there was no elevated health risk associated with playing on synthetic turf pitches. Currently, there is very limited evidence of an association between synthetic turf use and adverse health outcomes. Considering the ubiquitous use of synthetic grass globally and the scarcity of epidemiological studies, there is a vital need for further research based on longitudinal study designs and more robust exposure assessments, to help improve our understanding of any potential health risks associated with synthetic turf infill exposures.

## 1. Introduction

The use of synthetic turf in sporting fields has grown significantly in recent years, offering several advantages compared to natural grass, including lower maintenance costs due to reduced water and gardening requirements. Synthetic turf features a multilayer design composed of various materials. The synthetic grass fibres originate from polypropylene (PP), polyethylene (PE), and nylon, where structural support, cushioning, and traction for the synthetic grass fibres are provided by an infill layer. This layer often consists of rubber crumb from recycled tyres or specially manufactured rubber or cork. Beneath this, backing layers made of PP, polyurethane (PU), or latex, followed by a sand and gravel foundation, offer additional reinforcement [1]. Despite the economic appeal of synthetic grass, growing health concerns are emerging regarding the potential health risks associated with artificial turf, particularly the components of rubber crumb infill [2,3,4]. The environmental impact should also not be overlooked, considering artificial turf does not provide an ecological habitat, promote soil health, or reduce stormwater runoff like natural grass. Furthermore, the manufacturing, transportation and installation of artificial turf adds to our carbon footprint [5].

**Figure 1:**
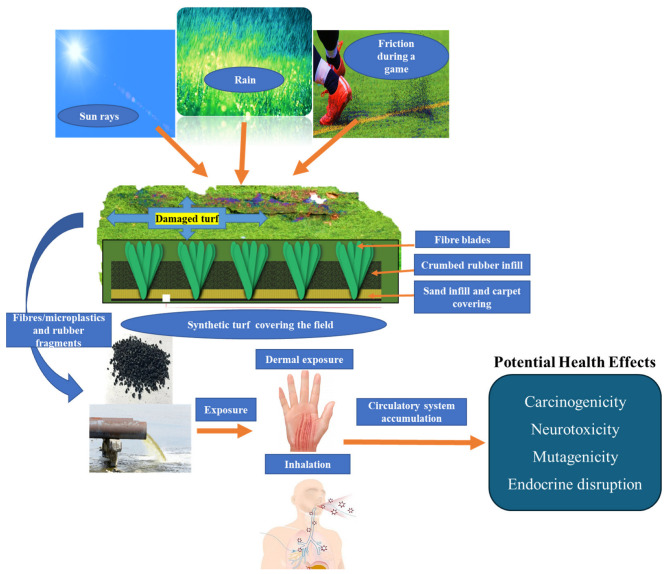
Degradation of synthetic turf in the environment and its exposure pathways.

Rubber crumb infill contains various hazardous contaminants, including volatile and semi-volatile organic compounds (VOCs and SVOCs) and metal(loids), which can leach into the environment and the air as the crumb rubber disintegrates over time through natural weathering or usage. The release of these compounds is influenced by environmental stressors (such as UV radiation, temperature, and mechanical stress) [6]. Consequently, athletes and members of the community engaging in recreational activities on synthetic sporting fields may unknowingly be exposed to potentially harmful chemicals. Exposure to contaminants from synthetic turf with rubber crumb can occur through inhalation, ingestion, or dermal contact (Figure 1). Dermal contact and inhalation are reported as the primary exposure routes for VOCs, such as polycyclic aromatic hydrocarbons (PAHs) and other chemicals released from synthetic turf [7,8].

A higher respiratory rate during physical activity may also lead to enhanced inhalation or even ingestion of rubber infill constituents as they become airborne. This risk is particularly serious in children who have greater ventilation compared to adults [9]. Notably, exposure to the constituents of tyre rubber has previously been linked to increased risk of leukaemia, lymphoma, and cancers of the urinary bladder and stomach in rubber manufacturing settings [10,11,12,13].

Given the ubiquitous use of synthetic turf with rubber crumb infill today, it is important to understand the extent of exposure to contaminants released from synthetic turf with rubber infill and the potential health impacts on users [14]. This review aims to assess the existing literature concerning exposure to PAHs and metal(loids) derived from rubber infill found in synthetic turf and their impact on health and provide recommendations for future studies.

## 2. Methods

### 2.1. Definitions and Scope

For purposes of this review, artificial turf or synthetic grass was defined as any surface comprised of synthetic fibres made to resemble grass with a cushioning layer composed of rubber crumb infill followed by a polyurethane or latex base layer, used in residential landscaping applications, playgrounds, or as a playing surface in sporting fields.

### 2.2. Literature Search

A comprehensive database search was conducted by S.K and I.P. for English-language, peer-reviewed, epidemiological and toxicological laboratory studies (animal and in vitro), as well as government reports published up to 2 December 2024. We searched Google Scholar, PubMed, Web of Science, Scopus, and Embase using keywords listed in Supplementary Table S1. We also searched reference lists of included articles. Duplicate studies identified in more than one database were excluded.

Due to the limited availability of epidemiological studies on this topic, a systematic review of such studies was not possible. Therefore, this review draws upon various other sources of evidence, including laboratory studies and government reports, to explore potential chemical exposures related to synthetic turf rubber crumb infill.

### 2.3. Inclusion Criteria

We screened the title and abstract of the identified studies to see if they examined human and/or animal chemical exposures related to synthetic turf and related exposure risks or evaluated either the PAH, VOC, SVOC, and metal(loids) concentrations in recycled rubber infill, potential air emissions from recycled rubber, or health impacts of chemicals from exposure (including occupational exposure, which may occur during installation, maintenance, and disposal of artificial turf) to recycled rubber infill.

Occupational studies on rubber manufacturing were not included in this review as they present different exposure risks that primarily occur during rubber processing (raw materials handling, milling of uncured rubber, extruding to form rubber sheeting, curing, and vulcanising) [15]. Publications based on musculoskeletal or burn injuries were excluded. Studies published in a language other than English or occupational studies on rubber manufacturing were excluded.

## 3. Results

### 3.1. Laboratory Studies on Rubber Crumb Infill

#### 3.1.1. PAH Levels in Rubber Crumb Infill

Eighteen laboratory studies investigated concentrations of PAHs found in synthetic turf rubber crumb infill (Table 1). Twelve of the studies were conducted in Europe, two in the United States of America (USA), two in Japan, one in China, and one in Egypt. In all the reviewed studies, rubber crumb infill samples were collected directly from playing fields, then bottled and refrigerated until laboratory examination. The samples were typically prepared for chemical analysis in fume cupboards covered with aluminium to prevent photo-degradation and contamination. Gas chromatography-mass spectrometry was the primary analytical technique used in most studies (*n* = 15) to analyse the PAH levels, while two studies utilised high-performance liquid chromatography [16,17]. The number of PAHs analysed in each study varied between eight and forty-six PAHs.

Overall, there was a high level of heterogeneity in the PAH concentrations found in rubber crumb samples. For example, the total PAH levels ranged from 0.4 mg/kg (median: 20.5 mg/kg) in the U.S. study by Zhang, Han, Zhang and Crain [17] to 3196 mg/kg (median: 2836 mg/kg) in the Egyptian study by Mohammed, et al. [18]. Two studies also highlighted that the total PAH concentrations in rubber crumb infill were directly influenced by the age of the synthetic turf [16,17]. The older synthetic surface fields contained lower concentrations of PAHs than newly installed turfs unless rubber granules have been reapplied. In the study by Zhang, Han, Zhang and Crain [17], samples of rubber crumb infill from synthetic turf installed up to one year prior to analysis had total median PAH concentrations of 45.4 mg/kg (range: 20.5–58.2 mg/kg) compared to a median concentration of 19.5 mg/kg (range: 0.4–21.1 mg/kg) in artificial turf laid ≥ 2–7 years preceding the analysis. Similarly, the Italian study by Marsili, Coppola, Bianchi, Maltese, Bianchi and Fossi [16] found higher concentrations of PAHs in one-year-old synthetic turf (median 34.5 mg/kg; range: 9.1 to 58.2 mg/kg) relative to turf installed ≥2–8 years earlier (median: 8.3 mg/kg; range: 8–8.4 mg/kg).

The origin and type of rubber crumbs, whether derived from end-of-life tyres (coated or uncoated) or industrial synthetic rubber, had a significant impact on PAH concentrations. For example, in the Japanese study by Nishi, et al. [19], industrial rubber crumb-derived infill contained slightly lower levels of PAHs with a total concentration of 11.4 mg/kg (median: 4.4 mg/kg) compared to end-of-life tyre-acquired infill with a total PAH concentration of 14.7 mg/kg (median: 5.6 mg/kg). In the European study conducted across 14 countries by Schneider, et al. [20], uncoated, end-of-life tyre-derived infill contained a mean concentration of 51.1 mg/kg, which was almost four times greater than PAH levels found in uncoated infills made from non-end-of-life tyres, with a mean concentration of 16 mg/kg (mean 16 mg/kg). There was also a high level of variability in PAH concentrations in synthetic turf infill samples in the international study of 14 nations across four continents by Armada, Llompart, Celeiro, Garcia-Castro, Ratola, Dagnac and de Boer [13]. The highest total concentrations of PAHs were observed in infill samples from Chile (median: 19 mg/kg; range: 5.8–247 mg/kg), followed by Sweden (median: 38 mg/kg; range: 23–137 mg/kg). The lowest PAH levels were found in Italy (median: 7.1 mg/kg; range: 2.6–21 mg/kg) and Turkey (median: 8.5 mg/kg; range 7–13 mg/kg).

#### 3.1.2. PAH Levels in Air Samples Collected on Synthetic Turf Fields

Air samples collected on synthetic turf fields in the Italian study by Menichini, et al. [21] of two different fields found a total PAH concentration of 2.3 and 4 ng/m^3^ for the two sites, respectively. The other Italian study by Schilirò, et al. [22] of eight different synthetic turf fields reported a statistically significant difference (*p* = 0.01) between PAH concentrations found in air samples in summer (June) (range: 0.04–0.8 ng/m^3^; median: <0.09 ng/m^3^) compared to winter (November) (range: 5.5–13.3 ng/m^3^; median: <0.09 ng/m^3^).

#### 3.1.3. VOC Levels in Rubber Crumb Infill and Air

Two studies also investigated the total VOC concentrations in rubber crumb infill using gas chromatography-mass spectrometry [20,23]. Sakai, Tahara, Kubota, Kawakami, Inoue and Ikarashi [23] examined 27 VOCs in 46 different synthetic turf infill samples in Japan and observed total concentrations ranging from 1.7 to 38.2 mg/kg (median: 6.4 mg/kg). In the analysis of 11 VOCs by Schneider, de Hoogd, Madsen, Haxaire, Bierwisch and Kaiser [20], mean VOC levels were higher in uncoated non-end-of-life tyre-derived infill (907.5 µg/m^3^; SD: 1004.6 µg/m^3^) compared to coated end-of-life tyre-containing infill (558 µg/m^3^; SD: 275.5 µg/m^3^). In air samples captured on three different sporting fields in Egypt, Mohammed, Saleh and Abdel-Latif [18] discovered VOC levels exceeding 400 mg/m^3^ (mean: 421 mg/m^3^; SD: 116 mg/m^3^).

#### 3.1.4. Metal(loids) Level in Rubber Crumb Infill

Six laboratory studies assessed the content of metal(loids) in rubber crumb infill (Table 1 [16,17,18,20,21,24]). Three out of the six studies were conducted in Italy [16,21,24], one was a transnational study across 14 European countries [20], whereas the remaining two studies were from the USA [17] and Egypt [18]. Overall, rubber granulate showed high variability in the metal(loids’) composition, with the highest median concentrations reported for Aluminium (5382 mg/kg), Zinc (5165 mg/kg), and Iron (489.6 mg/kg), followed by Cobalt (69.5 mg/kg), Lead (26.2 mg/kg), Nickel (26.1 mg/kg), Copper (14 mg/kg), Chromium (5.1 mg/kg), Cadmium (1.88 mg/kg), and Arsenic (0.9 mg/kg). The uncoated end-of-life tyre-derived rubber crumb infill had higher median concentrations of heavy metals (Aluminium 5382 mg/kg; Zinc 5165 mg/kg; Iron 489.6 mg/kg) compared to coated end-of-life tyre infill (Aluminium 3322.3 mg/kg; Zinc 2063 mg/kg; Iron 201 mg/kg). There was negligible variability in the metal content of rubber crumb infill samples from synthetic turf installed 1–8 years prior to testing (Zinc 5017 mg/kg; Iron 444.9 mg/kg; Copper 20.9 mg/kg) compared to newly laid turf (Zinc 5314 mg/kg; Iron 444.9 mg/kg; Copper 20.9 mg/kg). Similarly, there were minor differences in the median metal concentrations measured in rubber crumb infill samples from synthetic turf exposed to direct sunlight (Aluminium 499 mg/kg; Zinc 51 mg/kg; Iron 1084 mg/kg; Copper 14 mg/kg; Lead 43 mg/kg) compared to turf used in indoor sporting fields (Aluminium 412 mg/kg; Zinc 58 mg/kg; Iron 918 mg/kg; Copper 14 mg/kg; Lead 43 mg/kg).

**Table 1:** Summary of laboratory studies examining PAH, SVOC, VOC, and metal(loid) concentrations in rubber crumb found in artificial turf and air samples collected at synthetic turf fields.

Study	Country	Synthetic Turf Samples (n)	Age of Synthetic Turf	Analysis	Key Findings
Armada, Llompart, Celeiro, Garcia-Castro, Ratola, Dagnac and de Boer [13]	17 countries in 4 continents	91	Not disclosed	Gas chromatography-mass spectrometry	Sum of all detected PAHs in each sample of synthetic turf in: Thailand (5 samples) ranged from 1.6 to 13 mg/kg (median: 9) Netherlands (5 samples) ranged from 14 to 25 mg/kg (median: 24) Italy (7 samples) ranged from 2.6 to 21 mg/kg (median: 7.1) Chile (11 samples) ranged from 5.8 to 247 mg/kg (median: 19) Croatia (2 samples) ranged from 20 to 43 mg/kg (median: 33) Finland (10 samples) ranged from 1.6 to 13 mg/kg (median: 9.0) Turkey (2 samples) ranged from 7 to 13 mg/kg (median: 8.5) France (5 samples) ranged from 7.3 to 20 mg/kg (median: 14) Poland (3 samples) ranged from 12 to 87 mg/kg (median: 16) Greece (3 samples) ranged from 8.2 to 13 mg/kg (median: 11) Sweden (5 samples) ranged from 23 to 137 mg/kg (median: 38) Spain (7 samples) ranged from 8.2 to 42.4 mg/kg (median: 20) Canary Islands (4 samples) ranged from 0.048 to 19 mg/kg (median: 10) Portugal (5 samples) ranged from 8.9 to 40 mg/kg (median: 32)
Grynkiewicz-Bylina, Rakwic and Słomka-Słupik [10]	Poland	84	Not disclosed	Gas chromatography-mass spectrometry	Sum of all detected PAHs in each sample of synthetic turf median concentration 19.5 mg/kg (range: 15–26 mg/kg)
Bocca, Forte, Petrucci, Costantini and Izzo [24]	Italy	32	Not disclosed	Inductively coupled plasma optical emission spectroscopy	Metals Samples from 32 sporting fields: Al median concentration 755 mg/kg (min 1.2–6680 max mg/kg) Co median concentration 25 mg/kg (min 3.5–234 max mg/kg) Cr median concentration 6.2 mg/kg (min 0.4- 56 max mg/kg) Cu median concentration 12 mg/kg (min 0.8–60 max mg/kg) Fe median concentration 305 mg/kg (min 15–4318 max mg/kg) Ni median concentration 2 mg/kg (min 0.6–5.8 max mg/kg) Pb median concentration 22 mg/kg (min 12–46 max mg/kg) Zn median concentration 10,229 mg/kg (min 118–19,375 max mg/kg)
Celeiro, et al. [25]	Spain	15	6–8 years	Gas chromatography-mass spectrometry	Sum of all detected PAHs in each sample of synthetic turf ranged from 4.3 to 48.4 mg/kg (median: 19.9 mg/kg)
Celeiro, et al. [26]	Portugal	44 outdoor, 6 indoor	Not disclosed	Gas chromatography tandem mass spectrometry	Sum of all detected PAHs in each sample of synthetic turf ranged from 4.3 to 48.4 mg/kg (median: 17 mg/kg)
ECHA. [27]	Member States: Finland, Italy, the Netherlands, Portugal, and the United Kingdom	1373	Not disclosed	Not disclosed	Sum of all detected PAHs in new rubber granules manufactured from recycled tyres ranged from 9.12 to 58.21 mg/kg. Rubber granules from recycled tyres/SBR collected from fields ranged from 1.90 to 72.94 mg/kg Rubber granules from other recycled material (recycled scrap of vulcanised rubber and ground gaskets) ranged from 1.59 to 22.9 mg/kg
Gomes, et al. [28]	Portugal	3	New infill	Gas chromatography-mass spectrometry	Sum of all detected PAHs in each sample of synthetic turf ranged from 23.2 to 34.2 mg/kg
Ma, et al. [29]	China	64	Not disclosed	Gas chromatography-mass spectrometry	Sum of all detected PAHs in each sample of synthetic turf ranged from 0.045.15 to 1.08 mg/kg
Marsili, Coppola, Bianchi, Maltese, Bianchi and Fossi [16]	Italy	9	0–8 years	High-performance liquid chromatography-fluorescence	PAHs Sum of all detected PAHs in each sample of synthetic turf, median 16.1 mg/kg (range: 0.8–58.2 mg/kg). 6 samples of infill from synthetic turf installed 0–1 year prior, median concentration 34.5 mg/kg (range: 9.1–58.2 mg/kg) 3 samples of infill frum synthetic turf installed ≥2–8 years prior, median concentration 8.3 mg/kg (range: 8–8.4 mg/kg) Metals 5 samples of rubber crumb infill from newly installed synthetic turf: Cd median concentration 1.81 mg/kg (range: 0.47 to 2.68 mg/kg) Cr median concentration 4.12 mg/kg (range: 2.84 to 17.52 mg/kg) Cu median concentration 39.96 mg/kg (range: 5.59 to 84.49 mg/kg) Fe median concentration 489.60 mg/kg (range: 129.12 to 7256 mg/kg) Pb median concentration 17.51 mg/kg (range: 11.23 to 33.58 mg/kg) Ni median concentration 8.95 mg/kg (range: 4.11 to 26.12 mg/kg) Zn median concentration 5314 mg/kg (range: 3474 to 13,202 mg/kg) 4 samples of rubber crumb infill from synthetic turf installed 1–8 years prior: Cd median concentration 1.52 mg/kg (range: 0.47 to 2.38 mg/kg) Cr median concentration 3.26 mg/kg (range: 1.91 to 3.58 mg/kg) Cu median concentration 20.95 mg/kg (range: 5.49 to 65.11 mg/kg) Fe median concentration 444.90 mg/kg (range: 262.20 to 1577.40 mg/kg) Ni median concentration 5.25 mg/kg (range: 3.90 to 5.43 mg/kg) Pb median concentration 26.14 mg/kg (range: 10.76 to 38.99 mg/kg) Zn median concentration 5017 mg/kg (range: 4194 to 6006 mg/kg)
Menichini, Abate, Attias, De Luca, di Domenico, Fochi, Forte, Iacovella, Iamiceli, Izzo, Merli and Bocca [21]	Italy	13	Not disclosed	Gas chromatography coupled to low-resolution mass spectrometry	Sum of all detected PAHs over 2 sampling days concentration range 2.3–4 ng/m^3^
Menichini, Abate, Attias, De Luca, di Domenico, Fochi, Forte, Iacovella, Iamiceli, Izzo, Merli and Bocca [21]	Italy	13	Not disclosed	Gas chromatography coupled to low-resolution mass spectrometry	PAHs 2 samples of crumb rubber from coated recycled tyres (range: 1.90–28.5 mg/kg). 4 samples of crumb rubber from recycled uncoated tyres (range: 7.25–45.1 mg/kg). 2 samples of crumb rubber from recycled scrap of vulcanised rubber (range: 1.59–3.03 mg/kg). Metals 2 samples of crumb rubber from coated recycled tyres: Al concentration range 490–1028 mg/kg. Co concentration range 5.0–234 mg/kg. Copper (Cu) concentration range 12–60 mg/kg. Cr concentration range 1.8–6.2 mg/kg. Iron (Fe) concentration range 201–465 mg/kg Nickel (Ni) concentration range 0.67–5.8 mg/kg. Pb concentration range 0.7–28 mg/kg Zn concentration range 1063–19,375mg/kg. 4 samples of crumb rubber from recycled uncoated tyres: Al concentration range 164–755 mg/kg. Co concentration range 8.8–116 mg/kg. Cr concentration range 0.3–4.6 mg/kg. Cu concentration range 8.7–22 mg/kg. Fe concentration range 199–605 mg/kg Ni concentration range 1.3–2.5 mg/kg. Pb concentration range 12–26 mg/kg Zn concentration range 10,229–17,772 mg/kg. 2 samples of crumb rubber from recycled scrap of vulcanised rubber: Al concentration range 311–3260 mg/kg. Co concentration range 3.5–4.1 mg/kg. Cr concentration range 0.3–6.2 mg/kg. Cu concentration range 5.9–13 mg/kg. Fe concentration range 183–637 mg/kg. Ni concentration range 0.61–4.4 mg/kg. Pb concentration range 0.7–14 mg/kg. Zn concentration range 1408–7611 mg/kg.
Mohammed, Saleh and Abdel-Latif [18]	Egypt	15	New infill	Gas chromatography-mass spectrometry	PAHs Sum of all detected PAHs in each sample of synthetic turf ranged from 1802 to 3196 mg/kg (median: 2836). Metals New synthetic turf away from sunlight: Al mean concentration 412 mg/kg. Co mean concentration 20 mg/kg. Cu mean concentration 14 mg/kg. Cr mean concentration 58 mg/kg. Fe mean concentration 918 mg/kg. Ni mean concentration 36 mg/kg. Pb mean concentration 43 mg/kg Zn mean concentration 58 mg/kg. New synthetic turf exposed to sunlight Al mean concentration 499 mg/kg. Co mean concentration 14 mg/kg. Cu mean concentration 14 mg/kg. Cr mean concentration 51 mg/kg. Fe mean concentration 1084 mg/kg. Ni mean concentration 36 mg/kg. Pb mean concentration 43 mg/kg Zn mean concentration 51 mg/kg.
Mohammed, Saleh and Abdel-Latif [18]	Egypt	3	New infill	Gas chromatography-mass spectrometry	Monthly mean concentration 421 mg/m^3^ (SD 116 mg/m^3^)
Nishi, Kawakami, Sakai, Obama, Kubota, Inoue and Ikarashi [19]	Japan	10	Not disclosed	Gas chromatography-mass spectrometry	Sum of all detected PAHs in each sample of synthetic turf infill from: 24 samples of end-of-life tyre-derived infill 14.7 mg/kg (median: 5.6 mg/kg). 10 samples of industrial rubber-derived infill: 11.4 mg/kg (median: 4.4 mg/kg). 3 samples of infill with mixture/unknown composition: 10.6 mg/kg (median: 4.8 mg/kg).
Oomen and de Groot [30]	The Netherlands	100		Gas Chromatography-Mass Spectrometry	Sum of all detected PAHs in each sample of synthetic turf were detected up to 19.8 mg/kg (median: 5.8) VOC levels were all below LODs
Pavilonis, et al. [31]	USA	23	17 new infill and new fibre 7 filed sample ages: not disclosed	Gas chromatography-mass spectrometry	Sum of all detected PAHs were up to 2.75 mg/kg
Plesser and Lund [32]	Norway	6	Not disclosed	Not specified	Sum of all detected PAHs in each sample of synthetic turf ranged from 1 to 76 mg/kg
Ruffino, et al. [33]	Italy	5	1.5–3 years	Gas chromatography-mass spectrometry	Sum of all detected PAHs in each sample of synthetic turf median concentration 34.13 mg/kg (range: 3.45–61.81 mg/kg)
Sakai, Tahara, Kubota, Kawakami, Inoue and Ikarashi [23]	Japan	46	Not disclosed	Gas chromatography-mass spectrometry	Sum of all detected VOCs in each sample of synthetic turf, median 6.4 mg/kg (range: 1.7–38.2 mg/kg)
Schilirò, Traversi, Degan, Pignata, Alessandria, Scozia, Bono and Gilli [22]	Italy	6	Not disclosed	Gas chromatography coupled to low-resolution mass spectrometry	Sum of all detected PAHs: 7 sampling days in summer (June) median concentration < 0.09 ng/m^3^ (range: 0.04–0.8 ng/m^3^) 7 sampling days in winter (November) median concentration 5.9 ng/m^3^ (range: 5.5–13.3 ng/m^3^)
Schneider, de Hoogd, Madsen, Haxaire, Bierwisch and Kaiser [20]	14 European Countries	96	Not disclosed	PAHs: Gas chromatography-mass spectrometry and high-performance liquid chromatography. VOCs: Automated thermal desorption gas chromatography-mass spectrometry using non-polar HP-5 columns.	PAHs 47 samples of uncoated end-of-life tyre derived infill: mean concentration 51.1 mg/kg (standard deviation [SD] 18.7 mg/kg). 10 samples of coated end-of-life tyre derived infill: mean concentration 37.5 mg/kg (SD 7.7 mg/kg). 10 samples of uncoated non-end-of-life tyre infill: mean concentration 13.9 mg/kg (SD 16 mg/kg). VOCs (total concentration): 47 samples of uncoated end-of-life tyre derived infill: mean concentration 622.9 µg/m^3^ (SD1002.3 µg/m^3^). 10 samples of coated end-of-life tyre derived infill: mean concentration 558 µg/m^3^ (SD275.5 µg/m^3^). 10 samples of uncoated non-end-of-life tyre derived infill: mean concentration 907.5 µg/m^3^ (SD 1004.6 µg/m^3^). Metals 47 samples of uncoated end-of-life tyre derived infill: Aluminium (Al) mean concentration 5382.60 mg/kg (SD 10,107.7 mg/kg). Cd mean concentration 7.23 mg/kg (SD 12.60 mg/kg). Cobalt (Co) mean concentration 168.27 mg/kg (SD 101.30 mg/kg). Pb mean concentration 29.83 (SD 17.02 mg/kg). 10 samples of coated end-of-life tyre derived infill: Al mean concentration 3322.30 mg/kg (SD: 1886.40 mg/kg). Cd mean concentration 1.95 mg/kg (SD 1.36 mg/kg). Cobalt (Co) mean concentration 114.00 mg/kg (SD 54.65 mg/kg). Pb mean concentration 26.20 (SD 7.74 mg/kg). 10 samples of uncoated non-end-of-life tyre infill Al mean concentration 1249.70 mg/kg (SD 949.06 mg/kg). Cd mean concentration 0.25 mg/kg (SD 0.15 mg/kg). Cobalt (Co) mean concentration 11.45 mg/kg (SD 20.81 mg/kg). Pb mean concentration 9.40 (SD 11.31 mg/kg).
Zhang, Han, Zhang and Crain [17]	USA	8	~2 months–7 years	High-performance liquid chromatography	Metals In 5 samples of rubber granules from synthetic turf installed ≥2–7 years prior: Arsenic (As) median concentration 0.925 mg/kg (range: 0.28–3.55 mg/kg). Cadmium (Cd) median concentration 0.295 mg/kg (range: 0.21–0.41 mg/kg). Chromium (Cr) median concentration 0.93 mg/kg (range: 0.87–3.94 mg/kg). Lead (Pb) median concentration 4.63 mg/kg (range: 3.12–53.5 mg/kg). Zinc (Zn) median concentration 7849 mg/kg (range: 5710–9988 mg/kg).

One study also conducted leaching experiments by extracting 5 g of rubber crumb infill at room temperature for 24 h at pH 5, as well as in deionised water only for comparison [24]. The highest median leaching concentration of heavy metals was observed for samples leached in acidic conditions (pH 5) (Zinc 2456 µg/L; Lead 4.5 µg/L). In rubber crumb infill samples leached in deionised water, median heavy metal concentrations were significantly lower (Zinc 966 µg/L) and for some metals (Lead, Cadmium, Chromium, Mercury, Lead, and Tin) below detectable limits.

### 3.2. Epidemiological Studies on Rubber Crumb Infill Exposure

There were no epidemiological studies that directly examined exposure to rubber crumb infill in synthetic turf (including occupational exposure, which may occur during installation, maintenance, and disposal of artificial turf) and its associated adverse health outcomes. However, two studies have used proxy exposure measures to investigate the potential association between rubber crumb infill and haematological cancers (Table 2). One of the epidemiological studies, the American ecological study by Bleyer and Keegan [34], indirectly explored the association between rubber crumb infill exposure in synthetic turf and lymphoma. This study examined the synthetic turf field density (as a surrogate indicator of rubber crumb infill exposure) in relation to county-level lymphoma incidence in 58 counties in California between 2000 and 2013 [34]. The study found synthetic turf density, whether analysed as a categorical variable (low/intermediate/high) or as a continuous variable (number of synthetic turf fields per 100,000 population), was not significantly associated with county-level lymphoma incidence. The association remained statistically insignificant in sensitivity analyses stratified by counties with low or high median family income, ethnicity (non-Hispanic whites, Hispanics, Blacks, and Asians), and study period (2000–2013 and 2009–2013).

The other epidemiological study was a cross-sectional study by Wiesman and Lofy [35] in the United States. This study assessed the ratio of the observed-to-expected number of leukemia, non-Hodgkin lymphoma, and Hodgkin lymphoma cases among soccer players in Washington State relative to cancer rates in the general population between 2002 and 2015 [35]. The study used enrolment data from the Washington Youth Soccer Association covering records from 1983 to 2015 to estimate the number of individuals who had played soccer during the study period. They included individuals aged 6 to 24 years who had been diagnosed with cancer and had played soccer for at least 0.4 years whilst living in Washington during the study period. To estimate the total number of expected cancers among soccer players, the total person–years at risk for every soccer player that could have developed cancer were multiplied by the general population cancer rates in Washington State. The results identified that the ratio of observed cancer cases among Washington State soccer players (28 cases) relative to the expected number of cases (1384 cases) was 2% (95% CI 1–3%).

### 3.3. Metabolomic Studies on Rubber Crumb Infill Exposure

The level of 1-Hydroxypyrene in urine is widely utilised as a reliable urinary biomarker for exposure to PAHs. 1-Hydroxypyrene is a metabolite of pyrene, and its levels can range from less than 0.1 in individuals without exposure to 100 µmol/mol of creatinine in those with occupational exposure. The single metabolomic study conducted by [36] in the Netherlands enrolled seven healthy, non-smoking males to play a match of soccer on a synthetic turf containing recycled tyre crumb infill that had been reapplied two months prior [36]. The total exposure period was 2.5 h, which entailed 45 min of pre-match training and ground exercises and a match of 90 min in duration with a 15-min break to mimic heavy dermal contact involving sliding, sitting, and crawling on the playing surface. A total of 20 urine samples were obtained from each participant over a three-day period, including the day before the match, the day of the match, and one day after. The average hourly pre- and post-sporting event urinary elimination rate of 1-hydroxypyrne was examined using high-pressure liquid chromatography. Only one out of the seven participants excreted a statistically significant higher level of 1-hydroxypyrene (a PAH) (0.11 nmol/hour higher) post-match (0.14 nmol/hour) compared to pre-match (0.03 nmol/hour) levels. However, the researchers attributed this to concomitant dietary intake of PAHs rather than artificial turf field exposure [36].

### 3.4. Simulation Studies on Rubber Crumb Infill Exposure Using Human Body Fluids

Two studies simulated exposure to rubber crumb infill [31,37] (Table 2). The American study by Pavilonis, Weisel, Buckley and Lioy [31] simulated dermal, ingestion, and inhalation exposure to 16 different PAHs. The study suspended rubber crumb infill in synthetic solutions of biological fluids to mimic digestive, respiratory, and dermal exposure based on daily exposure of three hours over 130 days while considering body weight and surface area to body weight. The total PAHs exposure concentration estimated for all three exposure routes was 4.9 mg/kg. The study concluded that exposure to PAHs from rubber crumb infill occurred at concentrations that posed minimal to no health risks.

The other simulation study was the Dutch study by Pronk, Woutersen and Herremans [37]. The study also examined all three routes of exposure (dermal, ingestion, and inhalation) by first simulating the migration of rubber granulate into artificial sweat and gastrointestinal juices, as well as the evaporation of PAHs under warm conditions. The migration values derived in the laboratory analysis were then applied to exposure calculations (with input parameters for frequency and duration of exposure and body surface area) for different exposure scenarios, comparing field players and goalkeepers across different age groups. The study found that maximum PAHs exposure concentrations simulated for different exposure scenarios (ingestion: 1.78 mg/kg, dermal exposure: 0.004 mg/kg, and inhalation 19.8 mg/kg) did not exceed the European Union Toxicological Guidelines values for PAHs. Consequently, the study concluded that there was no elevated health risk associated with playing sports on synthetic turf pitches with rubber crumb infill.

### 3.5. Studies on Rubber Crumb Infill Exposure Using Animal Models

Three studies examined the potential toxicological effects of micronised rubber crumb infill using animal models [38,39,40]. LaPlaca and van den Hurk [39] exposed fish, including fathead minnow (*Pimephales promelas)* and mummichogs (*Fundulus heteroclitus*), to various concentrations of rubber crumb infill (micronised tyre fragments of 38–355 µm in size). The dissection of the fish following seven days of static renewal exposure (five fish per exposure of 0, 0.1, 0.3, 0.33, 1.0, and 6 g/L of micronised tyre fragments in 4 L glass jars containing aerated artificial seawater) showed a 40% mortality of fish exposed to the highest rubber crumb infill exposure concentration of 6 g/L. Bile fluorescence analysis found statistically significant higher levels of PAHs (2-, 4- and 5- ring structures) in fish exposed to 0.1 g/L, 0.3 g/L, 0.33 g/L, 1 g/L, and 6 g/L of micronised rubber crumb, compared to control group fish (*p* = <0.01). Similarly, liver enzyme activity (CYP1A) was also elevated in fish at all rubber crumb concentrations tested (*p* = <0.01). In their experiments of rubber crumb infill leachate, Cunningham et al. (2022) exposed zebrafish embryos (four *Daniro rerio* embryos and four *Daphnia magna* embryos) to suspensions of water containing micronised rubber crumb ranging from 0 to 3.0 × 10^9^ particles/mL or 0–100% leachate. After 48 h of exposure, zebrafish exposed to leachate of ≥80% developed abnormalities, including malformed jaws, snouts, and eyes, as well as yolk sac oedemas. There was also a significant increase in the total mortality to 45%. Similarly, in toxicological experiments by Xu, Lin, Cheong, Ridsdale, Tahara, Du, Das, Zhu, Peña Silva, Azimzada, Larsson and Tufenkji [40], physiological malformations, including stunted growth and impaired brain and cardiovascular system development, were observed in fertilised chicken eggs incubated in micronised crumb rubber infill water leachate.

## 4. Discussion

The reviewed studies highlighted that rubber crumb in synthetic turf contains various VOCs, SVOCs, and metal(loids), which may be released during use or through natural weathering as the synthetic turf deteriorates over time. To provide a scale of the issue, Kole, et al. [41] reported that an estimated 16,416 kg of crumb rubber is released into the environment on average per year in the US alone.

Our review demonstrated that SVOC, VOC, and metal(loid) levels in rubber crumb samples are influenced by the age of the synthetic turf, the origin and the type of rubber crumbs (end-of-life tyre-derived or industrial rubber) used. However, evidence of adverse health outcomes associated with exposure to rubber crumb infill remains limited and inconclusive. There is a scarcity of epidemiological studies examining direct exposure to rubber crumb infill and potential health effects. Although the two epidemiological studies we identified by Bleyer and Keegan [34],Wiesman and Lofy [35] found no significant association between exposure to rubber crumb infill and cancer incidence, they were not designed to detect causal relationships (both were cross-sectional studies) between synthetic turf exposure and specific health outcomes. Their population-level design also further limits the generalisability of their findings to individual-level health risks [17,31,37,42].

### 4.1. Limitations of Studies on Rubber Crumb Infill

It is important to recognise several limitations inherent in the available studies, underscoring the need for cautious interpretation of their findings. In addition to the small sample size, existing studies lack representative samples of diverse populations, making it challenging to generalise their results to broader contexts. This is particularly pertinent when considering children, as they may experience different exposure patterns and susceptibility compared to adults. Unfortunately, child health outcomes from synthetic turf exposure have not been extensively studied, leaving a significant gap in our understanding of potential risks for this vulnerable population. The lack of comprehensive investigations into how children interact with synthetic turf, including their play behaviours, duration of exposure, and unique vulnerabilities, poses a limitation in understanding the true extent of any health risks posed by such environments, especially long-term effects.

Whilst the simulation studies were insightful, they likely do not accurately reflect the bioavailability of contaminants within the human body, as simulation processes fail to account for toxicokinetic dynamics, including absorption, distribution, and elimination within a biological system. Furthermore, the understanding of the half-lives of these chemicals in the human body is incomplete, impeding comprehension of their elimination pathways and metabolic processes.

Discrepancies in measurement techniques and types of chemical compounds are also evident among the reviewed laboratory studies, further hampering cross-study comparisons. The varied laboratory standards across different countries hinder standardised assessments. Overall, these uncertainties underscore the complexity of establishing the safety of synthetic turf utilisation. It is, therefore, essential to approach the available evidence with caution while recognising the need for further rigorous research in this area.

### 4.2. Guidelines and Legislative Bodies Governing Use of Synthetic Grass

The popularity of synthetic turf extends globally, with several countries implementing guidelines and restrictions to mitigate exposure to contaminants from rubber infill in synthetic turf. Until the 2023 European Union (EU) ban on synthetic polymer microplastics (synthetic polymer particles < 5 mm of organic origin, insoluble, and non-biodegradable) in granular infill in synthetic sports fields, the total upper threshold limit for granules used in synthetic turf infill, for the key eight PAHs (identified as potential carcinogens), including Benzo[a]pyrene (BaP), Benzo[k]fluoranthene, Benzo[a]anthracene, Chrysene, Benzo[b]fluoranthene, Dibenzo[a,h]anthracene, and Indeno [1,2,3-cd]pyren) was 20 mg/g [43,44]. However, these EU limits are not universally adopted. In the Netherlands, for example, the upper limit of the eight PAHs was lower at 17 mg/g [45]. Other countries are yet to enforce threshold values for PAHs found in rubber crumb infill. As a result, there is a need for standardised monitoring, sampling methods, and threshold calculations to facilitate better cross-comparison and risk assessment.

Nine out of the eighteen laboratory studies examining rubber crumb infill reported total PAH concentrations exceeding the European guideline value of 20 mg/kg. The range of PAH concentrations was highly variable between studies, with levels ranging from 0.4 mg/kg to up to 3196 mg/kg. However, the age (natural weathering and wear and tear), type (end-of-life tyre vs. industrial rubber) of the synthetic turf infill, and whether it has been reapplied directly influence PAH concentrations and, therefore, the level of exposure that users are exposed to. Considering the number of laboratory studies that found PAH levels above the European (20 mg/kg) and Dutch (17 mg/kg) acceptable guidelines, this highlights the need for more government regulation and oversight to mitigate potential health concerns and ensure public safety. The two simulation studies that estimated the bioaccessibility of rubber crumb constituents in synthetic solutions of biological fluids to mimic digestive, respiratory, and dermal exposure found that the total PAH concentrations were below the guideline values (ranging between 0.004 mg/kg [dermal exposure] and 19.8 mg/kg [inhalation exposure]). The total PAH concentrations extracted from synthetic biofluids (digestive, dermal, and respiratory) were 4.9 mg/kg.

In terms of heavy metals, most countries (except for Italy) have no regulation stipulating acceptable metal concentrations in rubber crumb used as infill in synthetic sporting fields [46]. However, the median concentration of Zinc found in the leachate of samples extracted from synthetic turf in the Italian study by [24] significantly exceeded the acceptable Australian and New Zealand Environment and Conservation Council (ANZECC) guidelines for metals in soil and groundwater [47]. The median level for Zinc in this study (without acidification) was 966 µg/L, which is greater than 100 times the recommended ANZECC level of Zinc in marine water, and for 99%, 95%, 90%, and 80%, the species protection was at 7 μg/L, 15 μg/L, 23 μg/L and 43 μg/L, respectively. Until further research can provide a clearer picture of the risks associated with the leaching of heavy metals from rubber crumb infill in synthetic turf into waterways, more stringent regulatory measures should be enforced as soon as possible to minimise potential impacts on environmental and human health.

### 4.3. Future Direction

The emphasis of future research should be more robust and longitudinal study designs coupled with the analysis of biological samples, such as blood and urine, to gauge the presence of PAHs, VOCs, and other contaminants in relation to health outcomes. Considering that children are more vulnerable to chemical exposures compared to adults due to their smaller size and body weight, developing physiology, and behavioural patterns (rolling, crawling, and hand-to-mouth interactions) [48], this necessitates the need for more focused investigations involving children.

Amidst the backdrop of escalating global temperatures attributed to climate change, the implications on outdoor sports and recreation, particularly on synthetic turf fields, gain prominence. Synthetic turf’s propensity to retain heat raises concerns, potentially affecting player safety and comfort [5]. This phenomenon could catalyse the release of compounds from infill materials during hot conditions, heightening exposure concerns. Elevated temperatures could also drive the evaporation of compounds, impacting both players and spectators.

## 5. Conclusions

Overall, there was very limited evidence of an association between synthetic turf use and adverse health outcomes. However, it is important to interpret these findings cautiously due to the scarcity of epidemiological studies. Notably, nine out of the eighteen laboratory studies we reviewed reported a total PAHs concentration exceeding the recommended European guideline values, which emphasised the need for more stringent government regulation of rubber crumb infill in synthetic turf. Further research with longitudinal study designs and more comprehensive exposure assessments with an emphasis on high-risk and more susceptible populations, particularly children, is urgently needed to improve our understanding of the potential health effects to which users of synthetic turf may unknowingly be exposed to.

## Figures and Tables

**Table 2:** Epidemiological, metabolic, and simulation studies on rubber crumb infill exposure.

Study	Country	Exposure Scenario	Study Population	Study Design	Outcomes Studied	Key Findings
Epidemiological studies						
Bleyer and Keegan [34]	USA	Playing on synthetic turf fields with varying density	lymphoma cases diagnosed between 14 and 30 years of age with first primary malignant Hodgkin or non-Hodgkin lymphoma.	Ecological study at county-level (n = 58 counties) during 2000 to 2013. Number of synthetic turf fields in each county was obtained from the Californian Environmental Protection Agency and defined as the number of fields per 100,000 population and expressed as low or high if the field density was above or below 8.5 synthetic fields per 100,000 population. County-level lymphoma incidence rates were calculated using National Cancer Institute SEER Program registry data. Regression analyses were conducted using ANOVA F-tests with additional sensitivity analyses stratified by study period (2009–2013 and 2000–2013), county population (<15,000 population), age distribution, synthetic turf field density groupings (<1, 1–7 and >7), and median family income.	County-level lymphoma incidence.	No statistically significant association was found between synthetic turf field density (as a categorical variable: low/intermediate/high, or as a continuous variable: per 100,000 population) and county-level lymphoma incidence during 2000 to 2013. County-level lymphoma incidence was also not found to be significantly associated with synthetic turf field density in sensitivity analyses stratified by counties with low or high median family income, ethnicity (non-Hispanic whites, Hispanics, Blacks, and Asians), or study period (2000–2013 and 2009–2013).
Wiesman and Lofy [35]	USA	Playing soccer on grass and artificial fields in	Washington State soccer players (6 to 24 years of age) who: Were diagnosed with cancer during 2002 to 2015. Played soccer for at least 0.4 years whilst living in Washington State before cancer diagnosis.	Cross-sectional study comparing observed and expected number of cancers during 2002 to 2015 in Washington State among soccer players vs. general population. Data from Washington Youth Soccer enrolment records from 1983 to 2015 were used to estimate the number of people who had played soccer during the study period. The total person–years at risk (every year a soccer player could have developed cancer from 2002 to 2015 contributed one person–year at risk) were multiplied by Washington State cancer rates to estimate the total number of expected cancers among soccer players.	Ratio of observed-to-expected number of leukemia, non-Hodgkin lymphoma, and Hodgkin lymphoma cases among soccer players in Washington State.	The ratio of observed cancers among Washington State soccer players (28 cases) relative to the number of expected (1384) was 2% (95% CI 1–3%). Specific cancer types, as well as goalkeepers and select/premier players, had lower than expected cancer cases.
Metabolomic studies						
van Rooij, GM, Jongeneelen and J [36]	Netherlands	Training and playing soccer on artificial turf fields.	Male nonsmoking soccer players (median age: 22 years, range: 21–31 years) who: Reported no use of coal tar-containing shampoo/soap or ointment. Were not employed in an occupation where exposure to PAHs may occur. Had no underlying skin disease.	Metabolomic study. Study subjects spent a total of 2.5 h on an outdoor synthetic playing surface (90 min match + 30 min training and ground exercises + 30 min warm and cool down). A total of 20 urine samples were obtained from each subject over a three-day period, including the day before the match, the day of the match, and one day after, and were examined using high-pressure liquid chromatography.	Hourly pre- and post-sporting event urinary elimination rate of 1-hydroxypyrne (a PAH).	Total PAHs concentration in rubber crumb infill of the turf used for the study was 24 mg/kg (rubber crumb infill had been reapplied two months prior to the study). Pre-sporting excretion rate ranged from 0.04 nmol/hour (SD 0.03) to 0.48 nmol/hour (SD 0.14). Post-sporting excretion rate ranged from 0.03 nmol/hour (SD 0.01) to 0.24 nmol/hour (SD 0.07). A statistically significant increase of 0.11 nmol/hour between post-sporting event (0.14 nmol/hour) and pre-sporting event (0.03 nmol/hour) in urinary elimination rate of 1-hydroxypyrene was observed in one of the seven subjects (*p =* 0.004).
Simulation studies						
Pavilonis, Weisel, Buckley and Lioy [31]	USA	Contact with synthetic turf products and infill materials in a controlled lab environment.	General population	Simulation study of three potential routes of exposure, including dermal, ingestion, and inhalation of PAHs in crumbed rubber tyre infill. Synthetic solutions were used as analogues of actual biological fluids (digestive, respiratory, and dermal). Ingestion exposure simulation: 200 mg of infill was suspended in 8 mL of artificial saliva and 100 mL of gastric fluid and shaken at 30 RPM (round per minute) for two hours at 37 degrees, after which half the extract was shaken again after adding 100 mL of intestinal fluid adjusted to a pH of 6.5 to simulate digestive processes. Dermal exposure simulation: 200 mg of infill was suspended in 20 mL of artificial sweat solution by shaking at 30 RPM in a water bath at 37 degrees for one hour. Inhalation exposure simulation: 100 mg of infill was suspended in 10 mL of synthetic lung solution by shaking at 30 RPM at 37 degrees for 24 h. PAH levels in all samples were analysed using direct solid phase microextraction followed by gas chromatography-mass spectrometry. Based on the analytical results, expected inhalation and ingestion exposure was simulated assuming three hours of exposure per day and 130 days per year, dependent on age and body weight. Dermal exposure was estimated using surface area to body weight.	Total oral, dermal, and inhalation exposure to 8 PAHs.	Total PAHs concentration extracted from synthetic biofluids (digestive, dermal, and respiratory): 4.9 mg/kg.
Pronk, Woutersen and Herremans [37]	Netherlands	Playing on synthetic turf pitches with rubber granulate	Amateur football players.	Simulation study of three potential routes of exposure, including dermal, ingestion, and inhalation to PAHs in crumbed rubber tyre infill. Artificial body fluids were used to simulate the migration of rubber granulate into artificial sweat and gastrointestinal juices, which could evaporate under warm conditions and result in inhalation. Dermal exposure simulation: Rubber granulate was covered with artificial sweat and left to stand in a Petri dish for 2 h at 37 degrees. Ingestion exposure simulation: An in vitro system (Tiny-TIM model) consisting of two compartments that simulate conditions in the stomach and small intestine was used whereby peristalsis was simulated for a total of 4 h at 37 degrees. Inhalation exposure simulation: Using headspace analysis, the evaporation of PAHs was evaluated by heating rubber granulate for 6 h at 60 degrees. PAH levels in all samples were then analysed by gas chromatography-mass spectrometry. The migration values derived in the laboratory analyses (dermal, inhalation, and ingestion) were then applied to exposure calculations for different exposure scenarios, comparing exposure between field players and goalkeepers across different age groups (4–11 years, 7 years, 11–18 years, and 18–35 years). The calculation input parameters considered frequency of exposure (hours per event and months per year) and uncovered body surface area in contact with surface (cm^2^). The simulated exposure levels were then compared to the European Union Toxicological Guidelines Values for PAHs.	Maximum oral, dermal, and inhalation exposure to 8 PAHs. The simulated exposure levels were then compared to the European Union Toxicological Guidelines Values for PAHs.	Maximum PAHs concentration simulated for different exposure scenarios: Oral exposure: 1.78 mg/kg Dermal exposure: 0.004 mg/kg Inhalation exposure: 19.8 mg/kg

## Supplementary Materials

The following supporting information can be downloaded at: https://www.mdpi.com/article/10.3390/epidemiologia6010004/s1, Table S1: Search strategy.
