# Supplementary material for: Rubber Crumb Infill in Synthetic Turf and Health Outcomes: A Review of the Literature on Polycyclic Aromatic Hydrocarbons and Metalloids
Source: Epidemiologia (Basel). 2025 Jan 25;6(1):4. doi: 10.3390/epidemiologia6010004 (PMC11843893; doi:10.3390/epidemiologia6010004)
Supplement: Supplementary file 1 [file epidemiologia-06-00004-s001.zip › epidemiologia-3309987-supplementary.pdf]

Target / Source	Search Terms	
**Synthetic turf**	“artificial turf” OR “Synthetic turf” OR “artificial grass” OR “rubber granulate infill” OR “synthetic pitches” OR “artificial pitch*” OR “synthetic grass” OR “synthetic playground surfaced” OR “hybrid turf” OR “astroturf”	
**Health implications**	“Health” OR “safety” OR “risk” OR “air quality” OR pollutant OR “chemical off-gassing” OR “particulates” OR “particulate matter” OR “toxic*” OR “VOC” OR “volatile gas*” OR “semi volatile organic compounds” OR “Polycyclic aromatic hydrocarbon*” OR “benzothiazole and methyl isobutyl ketone” OR “air pollution” OR “hazard*”	
**PubMed**	("Health"[Title/Abstract] OR "safety"[Title/Abstract] OR "risk*"[Title/Abstract] OR "air quality"[Title/Abstract] OR "pollutant*"[Title/Abstract] OR "off-gassing"[Title/Abstract] OR "particulate*"[Title/Abstract] OR "toxic*"[Title/Abstract] OR "VOC"[Title/Abstract] OR "volatile gas*"[Title/Abstract] OR "semi volatile organic compound*"[Title/Abstract] OR "semi volatile organic compound*"[Title/Abstract] OR "volatile organic compound*"[Title/Abstract] OR "volatile organic compound*"[Title/Abstract] OR "polycyclic aromatic hydrocarbon*"[Title/Abstract] OR "benzothiazole"[Title/Abstract] OR "methyl isobutyl ketone"[Title/Abstract] OR "air pollution"[Title/Abstract] OR "Particle pollution"[Title/Abstract] OR "hazard*"[Title/Abstract] OR ("environmental exposure"[MeSH Terms] OR "hazardous substances"[MeSH Terms] OR "Health"[MeSH Terms] OR "toxic actions"[MeSH Terms] OR "Environmental Pollutants"[MeSH Terms] OR "Environmental Pollutants"[Pharmacological Action])) AND ("artificial turf"[All Fields] OR "Synthetic turf"[All Fields] OR "artificial grass"[All Fields] OR "rubber granulate infill"[All Fields] OR "synthetic pitch*"[All Fields] OR "artificial pitch*"[All Fields] OR "synthetic grass"[All Fields] OR "synthetic playground surfac*"[All Fields] OR "hybrid grass"[All Fields] OR "astroturf"[All Fields])	N=191
**Scopus search**	(TITLE-ABS-KEY ("artificial turf" OR "Synthetic turf" OR "artificial grass" OR "rubber granulate infill" OR "synthetic pitch*" OR "artificial pitch*" OR "synthetic grass" OR "synthetic playground surfac*" OR "hybrid turf" OR "hybrid grass" OR "astroturf") AND TITLE-ABS-KEY ("Health" OR "safety" OR "risk*" OR "air quality" OR pollutant* OR "off-gassing" OR "particulate*" OR "toxic*" OR "VOC" OR "volatile gas*" OR "semi volatile organic compound*" OR "semi-volatile organic compound*" OR "Volatile Organic Compound*" OR "Volatile-Organic Compound*" OR "Polycyclic aromatic hydrocarbon*" OR "benzothiazole" OR "methyl isobutyl ketone" OR "air pollution" OR "Particle pollution" OR "hazard*") AND NOT TITLE-ABS-KEY (injur*))	N=209
**Embase search**	('artificial turf'/exp OR 'artificial turf' OR 'synthetic turf' OR 'artificial grass' OR 'rubber granulate infill' OR 'synthetic pitch*' OR 'artificial pitch*' OR 'synthetic grass' OR 'synthetic playground surfac*' OR 'hybrid turf' OR 'hybrid grass' OR 'astroturf') AND ('health'/exp OR 'health' OR 'safety'/exp OR 'safety' OR 'risk*' OR 'air quality'/exp OR 'air quality' OR pollutant* OR 'off-gassing' OR 'particulate*' OR 'toxic*' OR 'voc' OR 'volatile gas*' OR 'semi volatile organic compound*' OR 'semi-volatile organic compound*' OR 'volatile organic compound*' OR 'volatile-organic compound*' OR 'polycyclic aromatic hydrocarbon*' OR 'benzothiazole'/exp OR 'benzothiazole' OR 'methyl isobutyl ketone'/exp OR 'methyl isobutyl ketone' OR 'air pollution'/exp OR 'air pollution' OR 'particle pollution' OR 'hazard*') NOT injur*	N=268
**CINAHL**	TI (“Health” OR “safety” OR “risk*” OR “air quality” OR pollutant* OR “off-gassing” OR “particulate*” OR “toxic*” OR “VOC” OR “volatile gas*” OR “semi volatile organic compound*” OR “semi-volatile organic	N=80

	compound*” OR “Volatile Organic Compound*” OR “Volatile-Organic Compound*” OR “Polycyclic aromatic hydrocarbon*” OR “benzothiazole” OR “methyl isobutyl ketone” OR “air pollution” OR “Particle pollution” OR “hazard*”) OR AB (“Health” OR “safety” OR “risk*” OR “air quality” OR pollutant* OR “off-gassing” OR “particulate*” OR “toxic*” OR “VOC” OR “volatile gas*” OR “semi volatile organic compound*” OR “semi-volatile organic compound*” OR “Volatile Organic Compound*” OR “Volatile-Organic Compound*” OR “Polycyclic aromatic hydrocarbon*” OR “benzothiazole” OR “methyl isobutyl ketone” OR “air pollution” OR “Particle pollution” OR “hazard*”)<br>OR<br>(MH "Health") OR (MM "Environmental Pollution+") OR (MH "Hazardous Materials")	
